# Identification of Specific Oral and Gut Pathogens in Full Thickness Colon of Colitis Patients: Implications for Colon Motility

**DOI:** 10.3389/fmicb.2018.03220

**Published:** 2019-01-04

**Authors:** Vasudevan Dinakaran, Sammed N. Mandape, Kristina Shuba, Siddharth Pratap, Shruti S. Sakhare, Mohammad Ali Tabatabai, Duane T. Smoot, Cherae M. Farmer-Dixon, Lakshmyya N. Kesavalu, Samuel Evans Adunyah, Janet Hayes Southerland, Pandu R. Gangula

**Affiliations:** ^1^Department of ODS & Research, School of Dentistry, Meharry Medical College, Nashville, TN, United States; ^2^Bioinformatics Core, School of Graduate Studies/Research & School of Medicine, Meharry Medical College, Nashville, TN, United States; ^3^Department of Public Health, School of Graduate Studies & Research, Meharry Medical College, Nashville, TN, United States; ^4^Department of Internal Medicine, Division of Gastroenterology & Hepatology, Meharry Medical College, Nashville, TN, United States; ^5^Department of Periodontology, College of Dentistry, University of Florida, Gainesville, FL, United States; ^6^Department of Biochemistry, Cancer Biology, Neuroscience & Pharmacology, Meharry Medical College, Nashville, TN, United States; ^7^Department of Nutrition Metabolism & Oral Surgery, University of Texas Medical Branch at Galveston, Galveston, TX, United States

**Keywords:** colitis, colon motility, nitric oxide (NO), antioxidants, oral microbiome, operational taxonomic units (OTUs), gut microbiome

## Abstract

Impaired colon motility is one of the leading problems associated with inflammatory bowel disease (IBD). An expanding body of evidence supports the role of microbiome in normal gut function and in progression of IBD. The objective of this work is to determine whether diseased full thickness colon specimens, including the neuromuscular region (critical for colon motility function), contain specific oral and gut pathogens. In addition, we compared the differences in colon microbiome between Caucasians (CA) and African Americans (AA). Thirty-nine human full thickness colon (diseased colon and adjacent healthy colon) specimens were collected from Crohn's Colitis (CC) or Ulcerative Colitis (UC) patients while they underwent elective colon surgeries. We isolated and analyzed bacterial ribosomal RNA (rRNA) from colon specimens by amplicon sequencing of the 16S rRNA gene region. The microbiome proportions were quantified into Operational Taxonomic Units (OTUs) by analysis with Quantitative Insights Into Microbial ecology (QIIME) platform. Two hundred twenty-eight different bacterial species were identified by QIIME analysis. However, we could only decipher the species name of fifty-three bacteria. Our results show that proportion of non-detrimental bacteria in CC or UC colon samples were altered compared to adjacent healthy colon specimens. We further show, for the first time in full thickness colon specimens, that microbiome of CC and UC diseased specimens is dominated by putative oral pathogens belonging to the Phyla Firmicutes (*Streptococcus, Staphylococcus, Peptostreptococcus*), and Fusobacteria (*Fusobacterium*). In addition, we have identified patterns of differences in microbiome levels between CA and AA specimens with potential implications for health disparities research. Overall, our results suggest a significant association between oral and gut microbes in the modulation of colon motility in colitis patients.

## Introduction

Inflammatory bowel disease (IBD) is comprised of Crohn's disease / Crohn's colitis (CC) and Ulcerative colitis (UC). The term Colitis, refers to general inflammation of the inner lining of the colon arising from numerous underlying causes including idiopathic infection, IBD (either CC or UC), ischemic colitis, allergic reactions, and/or microscopic colitis. Distally, gingivitis, and periodontal disease are chronic inflammatory gum diseases associated with orange, red, yellow, purple, and green complex bacterial infections in sub-gingival areas of oral cavity (Popova et al., 2013).

Previous studies have shown that periodontal disease (PD) is a significant risk factor and contributor to many systemic diseases, including IBD (Vavricka et al., 2013). Several factors including genetic, dietary, and environmental factors could influence the pathogenesis of microbiome (oral and gut) which in turn may increase the incidence of periodontitis and IBD (Lira-Junior and Figueredo, 2016; Agossa et al., 2017). In addition, *Porphyromonas gingivalis* known to cause PD altered the gut microbiota leading to increased gut epithelial permeability and endotoxemia, which causes systemic inflammation (Hajishengallis, 2015). In addition, many earlier studies have shown intestinal colonization of oral bacteria in the pathogenesis of IBD (Strauss et al., 2011; Atarashi et al., 2017).

Innumerable number of studies have shown that the gut microbiome including Phyla Proteobacteria, Firmicutes, and Bacteroidetes contribute to normal gut function (Mariat et al., 2009; Koliada et al., 2017; Walker et al., 2018; Zhao et al., 2018). Colon motility is mainly regulated by neuromuscular portion of the colon and this was shown to be impaired in colitis patients; putatively due to a reduction in neuronal nitric oxide (NO) synthase (nNOS) protein expression and/or neuronal degeneration (Bassotti et al., 2014; Gangula et al., 2017). Previous studies have analyzed the microbiome in feces and/or colon mucosal biopsy specimens of colitis patients (Gibson et al., 1991; Bibiloni et al., 2006). However, the relationship/interaction between oral and gut bacteria in the development and/or exacerbation of inflammatory disease in the colon (containing neuromuscular tissue) was under studied. In addition, data is limited on how oral bacteria interact with and influence the large intestinal flora, thereby contributing to colitis. Since motility of the colon is impaired in colitis patients and neuromuscular tissue play a role in the motility function (Geboes and Collins, 1998; Poli et al., 2001), we hypothesize that the interaction between oral and gut microbiome may play a significant role in the inflammatory processes associated with the development and progression of colitis seen in certain patient populations. Furthermore, we hypothesize that difference in microbiome may exist between CA and AA colitis patients, potentially contributing to health disparities in IBD.

## Methods

### Ethics Statement

The participants provided both written and verbal informed consent to Collaborative Human Tissue Networking (CHTN) Consortium to collect specimens while they underwent elective colon surgeries.

### Collection of Specimens

Frozen full thickness colon specimens were obtained from Cooperative Human Tissue Networking (CHTN). Thirty-nine human full thickness colon (moderate to severe diseased colon and adjacent healthy colon) specimens were collected from CC and UC male and female patients (ages between 18 and 75 years old) while they underwent elective colon surgeries. The specimens include Ulcerative (*n* = 13), Crohn's (*n* = 13) and adjacent healthy (*n* = 13) specimens. Characteristics of participants included Caucasians (CA) (*n* = 30) and African Americans (AA) (*n* = 9). CC male and female patients presented with symptoms like fever, fatigue, diarrhea, blood in stool, mouth sores, abdominal cramping, and pain around the anus, reduced appetite, and weight loss. While UC male and female patients presented with additional signs like rectal pain, rectal bleeding, and inability to defecate despite urgency.

### Extraction of DNA, Amplification of 16S rRNA Gene and Amplicon Sequencing

DNA extraction and microbial analysis were performed in the University of North Carolina at Chapel Hill School of Medicine Microbiome Core Facility (UNC: MC). We identified a conserved region of the 16S rRNA gene of 550 bp to amplify. This encompassed variable regions V3–V4 from the colon genomic DNA using primers 16S rRNA-F 5′-AGAGTTTGATCCTGGCTCAG-3′and 16S rRNA-R 5′-GCTGCCTCCCGTAGGAGT-3′ and overhang adapter sequences appended to the primer pair for compatibility with Illumina index and sequencing adapters. Briefly, each 16SrRNA amplicon was purified using AMPure XP reagent (Beckman Coulter, Indianapolis, IN, USA). Specifically, each sample was amplified using a limited cycle PCR program, adding Illumina sequencing adapters and optional dual-index barcodes [index 1(i7) and index 2(i5)] (Illumina, San Diego, CA, USA) to the amplicon target. The final libraries were purified using AMPure XP reagent, quantified and normalized prior to pooling. The DNA library pool was denatured with NaOH, diluted with hybridization buffer and heat denatured before loading on to the MiSeq reagent cartridge and to the MiSeq instrument (Illumina). The standard Illumina paired-end 250 base pair (PE250) protocol was used for sequencing the16S rRNA amplicons (Illumina, CA, USA).

### Processing of Sequence Reads

**Figure 1:**
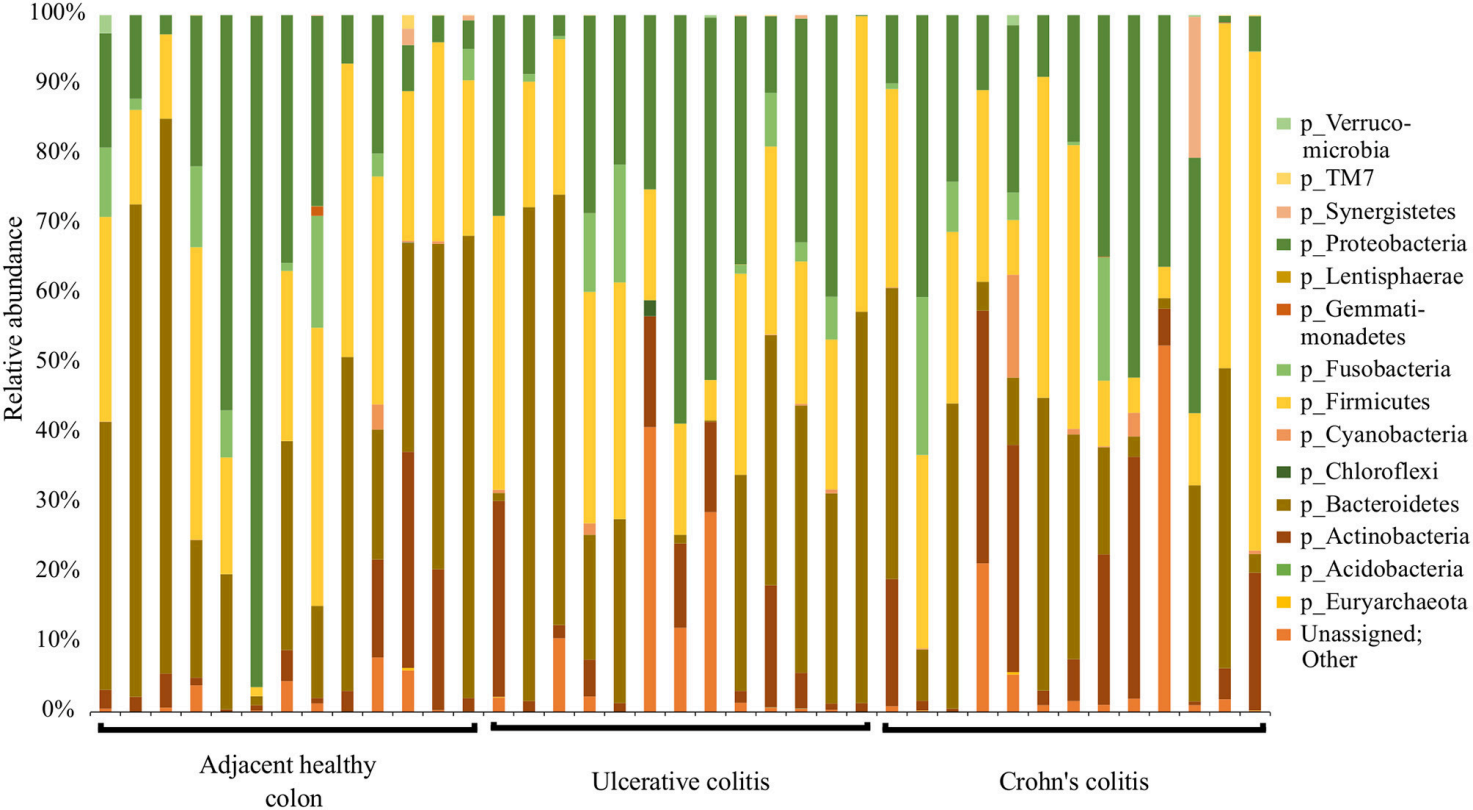
Summary of major bacterial taxa contributing to the communities detected in the 39 specimens at the Phylum level in the colitis and adjacent healthy specimen groups. Specimens are categorized into adjacent healthy colon (*n* = 13), Ulcerative colitis (UC, *n* = 13), and Crohn's colitis (CC, *n* = 13). Data represented are the relative abundances (%) of each phylum identified in each specimen.

Data was analyzed and microbial proportions using Operational Taxonomic Units (OTUs) were determined using Quantitative Insights Into Microbial ecology (QIIME) pipeline (Caporaso et al., 2010a) in the Meharry Medical College Bioinformatics Core. Briefly, generated raw reads were preprocessed for adapter removal. Processed sequence reads were obtained as fastq files and were converted into fasta, quality and flow files using Mothur package (Schloss et al., 2009). The initial number of fasta sequences obtained were 31,09,793. First, the fasta files were cleaned of host reads by mapping on to 9 mm mouse genome. Then, the primer sequences and barcode sequences were removed, demultiplexed and quality filtered. The number of high quality sequences remaining after quality filtering was 16,64,769. The OTUs were picked by *de novo* strategy. The high quality sequences were clustered at 97% identity using UCLUST inbuilt in QIIME pipeline to generate 3994 OTUs and taxonomy was assigned to OTU representative sequences using UCLUST (Edgar, 2010). The picked sequences were aligned using PyNAST aligner (Caporaso et al., 2010b). The chimeric sequences and singleton OTUs were removed using ChimeraSlayer (Haas et al., 2011). We constructed a phylogenetic tree for the sequences using FastTree version 2.1.3 (data not shown) (Price et al., 2010). Next, an OTU table was constructed and taxa were summarized using the 894 OTUs obtained from QIIME pipeline. α-diversity metrics was computed using Chao1 (abundance-based richness estimator) and Shannon analysis (diversity index) and Rarefaction plots were constructed (data not shown). β-diversity metrics was computed using weighted and unweighted Principal Coordinates Analysis (PCoA) (data not shown) (Gower, 2005). A Taxonomic Summary Bar plot showing OTUs assigned to Phyla-level taxonomy per sample was subsequently constructed (Figure 1). Bar Plots showing the relative abundance of bacteria at the Phyla-level between races, diseased tissue and healthy tissue groups is shown in **Figure 3**. Sample-specific sequences were deposited in the MGRAST database (accession number: b3b851ba2c6d676d343739393937332e33) and was assigned an MG-RAST project ID (mgs675214) (Keegan et al., 2016). In addition, sample-specific sequences were deposited in the NCBI (BioProject: PRJNA496071).

The pathogenic and beneficial oral and gut bacteria were identified using the NCBI Genome database (https://www.ncbi.nlm.nih.gov/genome/). This analysis was performed to assess the pathogenic and healthy bacterial proportions in human full thickness colon specimens (Tables 1–3).

**Table 1:** Functions and proportions of specific pathogenic Oral bacteria colonized in full thickness colon specimens.

**Sl. No**.	**Bacteria genus**	**Bacteria species**	**Proportion (%)**	**Bacteria phylum**	**Function in IBD**	**NCBI genome database link**
**ADJACENT HEALTHY COLON**						
1	Prevotella	stercorea	2.3	Bacteroidetes	Alters mucosal microbiota in the colon of patients with IBD	H
2	Prevotella	Other	0.3	Bacteroidetes	A microbial signature of Crohn's disease	GS
3	Gemella	s__	0.1	Firmicutes	Microbiome in New-Onset Crohn's Disease	CP
4	Staphylococcus	sciuri	0.1	Firmicutes	Develops intestinal inflammation in acute and chronic colitis	I
5	Staphylococcus	aureus	0.6	Firmicutes	Causes Crohn's disease	AU
6	Abiotrophia	s__	0.1	Firmicutes	Causes fecal microbial dysbiosis in IBD	CS
7	Lactobacillus	zeae	1.9	Firmicutes	Maintains remission of ulcerative colitis	A
8	Lactobacillus	s__	0.4	Firmicutes	Maintains remission of ulcerative colitis	CW
9	Lactococcus	s__	0.7	Firmicutes	Used in the treatment of Crohn's disease	CX
10	Peptostreptococcus	anaerobius	11.6	Firmicutes	Causes dysbiosis in IBD	AW
11	Peptostreptococcus	s__	0.5	Firmicutes	Causes gut microbiota dysbiosis in IBD	DR
12	Selenomonas	s__	0.2	Firmicutes	Causes dysbiosis in colorectal cancer	EB
13	Eubacterium	dolichum	1.0	Firmicutes	Causes dysbiosis of the intestinal microbiota	AL
14	Fusobacterium	s__	2.2	Fusobacteria	Identified from colonic biopsies of IBD patients	EN
15	Pseudomonas	alcaligenes	1.8	Proteobacteria	Identified in the gut microbiota of IBD	AX
16	Pseudomonas	s__	0.1	Proteobacteria	Causes infection in Children with Early-onset Crohn's Disease	GG
17	Pseudomonas	Other	0.2	Proteobacteria	Gut microbe in children with early onset Crohn's disease	HR
18	Corynebacterium	durum	0.1	Actinobacteria	Gut microbe in IBD patients	AK
19	Corynebacterium	s__	0.8	Actinobacteria	Causes experimental colitis	BI
20	Pseudoramibacter_Eubacterium	s__	1.6	Firmicutes	Metabolizes Linoleic acid in the Gut	DF
**DISEASED COLON (ULCERATIVE COLITIS)**						
1	Prevotella	stercorea	1.0	Bacteroidetes	Alters mucosal microbiota in the colon of patients with IBD	H
2	Prevotella	s__	0.3	Bacteroidetes	A microbial signature of Crohn's disease	BZ
3	Prevotella	Other	0.3	Bacteroidetes	A microbial signature of Crohn's disease	GS
4	Staphylococcus	aureus	0.3	Firmicutes	Causes Crohn's disease	AU
5	Lactobacillus	zeae	7.6	Firmicutes	Maintains remission of ulcerative colitis	A
6	Lactobacillus	s__	0.3	Firmicutes	Maintains remission of ulcerative colitis	CW
7	Lactococcus	s__	0.6	Firmicutes	Used in the treatment of Crohn's disease	CX
8	Peptostreptococcus	anaerobius	12.7	Firmicutes	Causes dysbiosis in IBD	AW
9	Peptostreptococcus	s__	0.3	Firmicutes	Causes gut microbiota dysbiosis in IBD	DR
10	Selenomonas	s__	0.1	Firmicutes	Causes dysbiosis in colorectal cancer	EB
11	Eubacterium	dolichum	0.5	Firmicutes	Causes dysbiosis of the intestinal microbiota	AL
12	Pseudoramibacter_ Eubacterium	s__	1.9	Firmicutes	Metabolizes Linoleic acid in the Gut	DF
13	Fusobacterium	s__	3.0	Fusobacteria	Identified from colonic biopsies of IBD patients	EN
14	Pseudomonas	alcaligenes	0.4	Proteobacteria	Identified in the gut microbiota of IBD	AX
15	Pseudomonas	s__	0.8	Proteobacteria	Infection in Children with Early-onset Crohn's Disease	GG
16	Aggregatibacter	s__	1.4	Proteobacteria	Causes fungal microbiota dysbiosis in IBD	
17	Corynebacterium	s__	1.0	Actinobacteria	Causes experimental colitis	BI
**DISEASED COLON (CROHN'S COLITIS)**						
1	Prevotella	tannerae	0.2	Bacteroidetes	Prevalent in colitis	F
2	Prevotella	stercorea	3.3	Bacteroidetes	Alters mucosal microbiota in the colon of patients with IBD	H
3	Prevotella	melaninogenica	0.4	Bacteroidetes	Gut microbiome biomarker in ankylosing spondylitis	U
4	Prevotella	Other	3.3	Bacteroidetes	A microbial signature of Crohn's disease	GS
5	Gemella	s__	0.1	Firmicutes	Microbiome in New-Onset Crohn's Disease	CP
6	Staphylococcus	sciuri	0.1	Firmicutes	Develops intestinal inflammation in acute and chronic colitis	I
7	Staphylococcus	aureus	0.5	Firmicutes	Causes Crohn's disease	AU
8	Abiotrophia	s__	0.2	Firmicutes	Causes fecal microbial dysbiosis in IBD	CS
9	Lactobacillus	zeae	6.8	Firmicutes	Maintains remission of ulcerative colitis	A
10	Lactobacillus	reuteri	0.1	Firmicutes	Prevents colitis as a probiotic	M
11	Lactobacillus	s__	0.6	Firmicutes	Maintains remission of ulcerative colitis	CW
12	Lactococcus	s__	0.7	Firmicutes	Used in the treatment of Crohn's disease	CX
13	Peptostreptococcus	anaerobius	4.0	Firmicutes	Causes dysbiosis in IBD	AW
14	Peptostreptococcus	s__	0.1	Firmicutes	causes gut microbiota dysbiosis in IBD	DR
15	Selenomonas	s__	0.4	Firmicutes	Causes dysbiosis in colorectal cancer	EB
16	Eubacterium	dolichum	0.8	Firmicutes	Causes dysbiosis of the intestinal microbiota	AL
17	Pseudoramibacter_ Eubacterium	s__	1.3	Firmicutes	Metabolizes Linoleic acid in the Gut	DF
18	Fusobacterium	s__	2.4	Fusobacteria	Identified from colonic biopsies of IBD patients	EN
19	Pseudomonas	alcaligenes	0.8	Proteobacteria	Identified in the gut microbiota of IBD	AX
20	Pseudomonas	s__	1.0	Proteobacteria	Infection in Children with Early-onset Crohn's Disease	GG
21	Corynebacterium	durum	0.2	Actinobacteria	Gut microbe in IBD patients	AK
22	Corynebacterium	s__	0.1	Actinobacteria	Causes experimental colitis	BI
23	Pyramidobacter	piscolens	0.1	Synergistetes	Oral bacteria in IBD	P

*Specific Information of functions was adapted from NCBI Genome Database (https://www.ncbi.nlm.nih.gov/genome/)*.

*The bacterial species that could not be identified at the genus level are mentioned as g___ and the bacterial species that could not be identified at the species level are mentioned as s___*.

**Table 2:** Functions and proportions of specific beneficial Gut bacteria colonized in full thickness colon specimens.

**Sl. No**.	**Bacteria genus**	**Bacteria species**	**Proportion (%)**	**Bacteria phylum**	**Function in IBD**	**NCBI genome database link**
**ADJACENT HEALTHY COLON**						
1	Shuttleworthia	satelles	0.2	Firmicutes	Identified in the human ileum	J
2	Bifidobacterium	longum	0.1	Actinobacteria	Attenuates acute murine experimental model of IBD	Y
3	Rhizobium	leguminosarum	0.1	Proteobacteria	Identified commensal gut microbe	AB
4	Lysinibacillus	boronitolerans	12.1	Firmicutes	Identified commensal gut microbe	AT
5	Alloiococcus	s__	1.7	Firmicutes	Identified commensal gut microbe	CT
6	Christensenella	s__	2.2	Firmicutes	Identified gut microbe	DC
7	Blautia	s__	0.3	Firmicutes	Butyrate-producing bacterial species in Gut	DH
8	Coprococcus	s__	0.1	Firmicutes	Butyrate-producing bacterial species in Gut	DI
9	g__	s__	0.7	Gemmatimonadetes	Identified commensal gut microbe	EP
10	g__	s__	0.7	Lentisphaerae	Normal gut microbe	EQ
11	g__	s__	0.4	Proteobacteria	Identified commensal gut microbe	FC
12	Comamonas	s__	1.0	Proteobacteria	Identified commensal gut microbe	FL
13	Desulfovibrio	s__	0.3	Proteobacteria	Sulfate reducing bacteria in IBD	FV
14	Paracoccus	Other	0.1	Proteobacteria	Identified commensal gut microbe	HH
15	Other	Other	0.2	Proteobacteria	Mucosal and fecal microbe	HP
**DISEASED COLON (ULCERATIVE COLITIS)**						
1	Lysinibacillus	boronitolerans	8.2	Firmicutes	Identified commensal gut microbe	AT
2	Varibaculum	s__	0.2	Actinobacteria	Identified in the gut of a premature infant	BH
3	Alloiococcus	s__	3.1	Firmicutes	Identified commensal gut microbe	CT
4	Christensenella	s__	0.6	Firmicutes	Identified gut microbe	DC
5	Blautia	s__	0.3	Firmicutes	Butyrate-producing bacterial species in Gut	DH
6	Coprococcus	s__	0.2	Firmicutes	Butyrate-producing bacterial species in Gut	DI
7	g__	s__	0.3	Gemmatimonadetes	Identified commensal gut microbe	EP
8	g__	s__	0.3	Proteobacteria	Identified commensal gut microbe	FC
9	Comamonas	s__	2.7	Proteobacteria	Identified commensal gut microbe	FL
10	Desulfovibrio	s__	0.1	Proteobacteria	Sulfate reducing bacteria in IBD	FV
11	Morganella	s__	0.1	Proteobacteria	Sulfate reducing bacteria in IBD	FZ
12	g__	s__	0.6	TM7	Identified commensal gut microbe	
13	Other	Other	0.1	Actinobacteria	Commensal gut bacteria in IBD	GN
14	Other	Other	0.5	Proteobacteria	Adult fecal microbe	HO
**DISEASED COLON (CROHN'S COLITIS)**						
1	Akkermansia	muciniphila	0.1	Verrucomicrobia	Adheres to enterocytes and strengthens the integrity of the epithelial cell layer	S
2	Bifidobacterium	longum	0.4	Actinobacteria	Attenuates acute murine experimental model of IBD	Y
3	Rhizobium	leguminosarum	0.2	Proteobacteria	Identified commensal gut microbe	AB
4	Anoxybacillus	kestanbolensis	0.3	Firmicutes	Identified commensal gut microbe	AD
5	Lysinibacillus	boronitolerans	11.5	Firmicutes	Identified commensal gut microbe	AT
6	g__	s__	0.1	Acidobacteria	Identified in human gut microbiota	BC
7	Varibaculum	s__	0.1	Actinobacteria	Identified in the gut of a premature infant	BH
8	SHD-231	s__	0.1	Chloroflexi	Identified in the fecal microbiome of Gout patients	CH
9	g__	s__	0.1	Cyanobacteria	Identified in human gut microbiota	CJ
10	Alloiococcus	s__	7.1	Firmicutes	Identified commensal gut microbe	CT
11	Christensenella	s__	0.1	Firmicutes	Identified gut microbe	DC
12	g__	s__	0.1	Firmicutes	Commensal gut bacteria in IBD	DG
13	Blautia	s__	0.1	Firmicutes	Butyrate-producing bacterial species in Gut	DH
14	Coprococcus	s__	0.1	Firmicutes	Butyrate-producing bacterial species in Gut	DI
15	g__	s__	0.3	Gemmatimonadetes	Identified commensal gut microbe	EP
16	g__	s__	0.3	Lentisphaerae	Normal gut microbe	EQ
17	Comamonas	s__	2.4	Proteobacteria	Identified commensal gut microbe	FL
18	g__	s__	0.1	Proteobacteria	Identified commensal gut microbe	FT
19	Desulfovibrio	s__	0.2	Proteobacteria	Sulfate reducing bacteria in IBD	FV
20	g__	s__	0.3	TM7	Identified commensal gut microbe	
21	Other	Other	0.2	Firmicutes	Commensal gut bacteria in IBD	HA
22	Paracoccus	Other	0.7	Proteobacteria	Identified commensal gut microbe	HH
23	Other	Other	0.1	Proteobacteria	Identified commensal gut microbe	HN
24	Other	Other	0.7	Proteobacteria	Adult fecal microbe	HO

*Specific Information of functions was adapted from NCBI Genome Database (https://www.ncbi.nlm.nih.gov/genome/)*.

*The bacterial species that could not be identified at the genus level are mentioned as g___ and the bacterial species that could not be identified at the species level are mentioned as s___*.

**Table 3:** Functions and proportions of specific pathogenic Gut bacteria colonized in full thickness colon specimens.

**Sl. No**.	**Bacteria genus**	**Bacteria species**	**Proportion (%)**	**Bacteria phylum**	**Function in IBD**	**NCBI Genome database link**
**ADJACENT HEALTHY COLON**						
1	Ochrobactrum	s__	0.1	Proteobacteria	Causes early bacterial dependent induction of inducible nitric oxide synthase (iNOS) in epithelial cells in experimental colitis	EU
2	Sphingomonas	s__	0.2	Proteobacteria	Tissue associated intestinal microflora	FF
3	Burkholderia	s__	1.2	Proteobacteria	causes dysfunction of GALT and gut flora in IBD	FI
4	Acinetobacter	rhizosphaerae	0.3	Proteobacteria	Identified gut bacteria in IBD	K
5	Acinetobacter	lwoffii	0.3	Proteobacteria	gut bacteria in multiple sclerosis patients	W
6	Stenotrophomonas	geniculata	1.2	Proteobacteria	Identified gut bacteria in IBD	AG
7	Staphylococcus	sciuri	0.1	Firmicutes	Develops intestinal inflammation in acute and chronic colitis	I
8	Staphylococcus	aureus	0.6	Firmicutes	Causes Crohn's disease	AU
9	Lactobacillus	zeae	1.9	Firmicutes	Maintains remission of ulcerative colitis	A
10	Lactobacillus	s__	0.4	Firmicutes	Maintains remission of ulcerative colitis	CW
11	Lactococcus	s__	0.7	Firmicutes	Used in the treatment of Crohn's disease	CX
12	Pseudomonas	alcaligenes	1.8	Proteobacteria	Identified in the gut microbiota of IBD	AX
13	Pseudomonas	s__	0.1	Proteobacteria	Infection in Children with Early-onset Crohn's Disease	GG
14	Pseudomonas	Other	0.2	Proteobacteria	Gut microbe in children with early onset Crohn's disease	HR
15	Bacillus	s__	0.2	Firmicutes	Increases cytokine levels in IBD	CL
16	Bacteroides	Other	0.1	Bacteroidetes	Commensal bacteria that induces colitis	GR
17	Microbacterium	maritypicum	0.1	Actinobacteria	Fecal microbiome in Obesity	V
18	Eggerthella	lenta	0.8	Actinobacteria	Causes bacteremia in Crohn's disease patient	AA
19	Brevundimonas	diminuta	0.1	Proteobacteria	Identified in the adult fecal microbiota of allergy patients	AO
20	Propionibacterium	acnes	5.3	Actinobacteria	Intestinal microbe in Liver disease	BA
21	Methanobrevibacter	s__	0.7	Euryarchaeota	Identified in the gut of IBD	BB
22	g__	s__	2.4	Acidobacteria	Identified in the gut microbiome of Type 2 Diabetes patients	BD
23	g__	s__	1.1	Actinobacteria	Identified in gut microbiota in IBD	BF
24	Actinomyces	s__	0.1	Actinobacteria	Identified in Abdominopelvic actinomycosis involving the GIT	BG
25	Varibaculum	s__	0.2	Actinobacteria	Identified in the gut of a premature infant	BH
26	Microbacterium	s__	0.1	Actinobacteria	Identified in the duodenum of children with ulcerative colitis	BK
27	g__	s__	0.2	Actinobacteria	Identified in fecal microbiota of pediatric IBD patients	BP
28	Atopobium	s__	0.1	Actinobacteria	Altered intestinal microbiota in Crohn's disease	BR
29	Slackia	s__	0.2	Actinobacteria	Human gut bacteria in Multiple Sclerosis	BS
30	g__	s__	0.1	Bacteroidetes	Characterized in intestinal biopsies in IBD patients	CB
31	g__	s__	0.1	Bacteroidetes	Human gut microbe in Obesity and IBD	CC
32	Cloacibacterium	s__	1.1	Bacteroidetes	Identified in the rectum of human colorectal adenoma patients	CG
33	g__	s__	0.2	Cyanobacteria	Identified in the gut microbiome of IBD patients	CI
34	g__	s__	0.6	Firmicutes	Causes microbiota dysbiosis in IBD	CO
35	g__	s__	0.7	Firmicutes	A microbial signature of Crohn's disease	DB
36	Clostridium	s__	0.9	Firmicutes	Causes infection of the gut in IBD	DE
37	Dorea	s__	0.1	Firmicutes	Causes dysfunction of the intestinal microbiome in IBD	DJ
38	Lachnospira	s__	0.1	Firmicutes	Gut bacteria in Crohn's disease patients	DK
39	Ruminococcus	s__	0.1	Firmicutes	Dominant in gut microbiome of IBD patients	DO
40	g__	s__	0.2	Firmicutes	Gut microbe in IBD	DP
41	g__	s__	0.6	Firmicutes	A microbial signature of Crohn's disease	DQ
42	Anaerotruncus	s__	0.2	Firmicutes	Tissue associated intestinal microflora	DT
43	Oscillospira	s__	0.4	Firmicutes	Gut microbe in IBD patients	DU
44	Ruminococcus	s__	0.7	Firmicutes	Dominant in gut microbiome of IBD patients	DV
45	g__	s__	0.4	Firmicutes	Gut microbe underlying the onset of IBD	DW
46	Acidaminococcus	s__	1.3	Firmicutes	Gut microbe in IBD	DX
47	Phascolarctobacterium	s__	1.9	Firmicutes	Causes dysfunction of the intestinal microbiome in IBD	DZ
48	Schwartzia	s__	0.5	Firmicutes	causes fecal microbial dysbiosis in IBD	EA
49	g__	s__	0.2	Firmicutes	A microbial signature of Crohn's disease	EC
50	Anaerococcus	s__	1.3	Firmicutes	Microbe in Inflammatory Pouch Complications	EE
51	Finegoldia	s__	0.3	Firmicutes	Intestinal microbe in colorectal cancer	EF
52	g__	s__	0.3	Firmicutes	Gut microbe in GI diseases	EI
53	Bulleidia	s__	0.1	Firmicutes	Fecal-associated and mucosalassociated microbiota in irritable bowel syndrome patients	EJ
54	Coprobacillus	s__	0.3	Firmicutes	Alters Gut Microbiota in Psoriatic Arthritis	EL
55	Leptotrichia	s__	0.7	Fusobacteria	Causes gut mucosal inflammation in Rheumatoid arthritis patients	EO
56	g__	s__	4.2	Proteobacteria	Intestinal microbe in children with severe and complicated acute viral gastroenteritis	EV
57	Methylobacterium	s__	0.1	Proteobacteria	Causes microbial dysbiosis in pediatric Crohn's disease	EW
58	g__	s__	0.1	Proteobacteria	Intestinal microbe in children with severe and complicated acute viral gastroenteritis	EX
59	g__	s__	1.2	Proteobacteria	Involved in host-microbial cross talk in IBD	FG
60	Lautropia	s__	0.3	Proteobacteria	causes fecal microbial dysbiosis in IBD	FJ
61	g__	s__	0.1	Proteobacteria	Fecal and mucosa associated microbe in IBD	FK
62	Citrobacter	s__	0.1	Proteobacteria	Gut microbe in newly diagnosed with treatment-naïve Crohn's disease patients	FY
63	Halomonas	s__	1.4	Proteobacteria	Intestinal microflora in chronic kidney disease	GB
64	g__	s__	0.1	Proteobacteria	Microbe in colon tissue from IBD subjects	GE
65	g__	s__	0.1	Proteobacteria	bacteria in human Ulcerative Colitis patients	GH
66	Other	Other	0.1	Actinobacteria	Alters fecal microbiota in pediatric IBD patients	GO
67	Other	Other	3.6	Firmicutes	gut microbe in experimental colitis	GT
68	Other	Other	12.5	Firmicutes	Fecal and mucosa associated microbe in IBD	GW
69	Weissella	Other	0.2	Firmicutes	Gut microbe in IBD patients	GX
70	Other	Other	0.1	Proteobacteria	Fecal and mucosa associated microbe in IBD	HL
71	Other	Other	0.1	Proteobacteria	Involved in host-microbial cross talk in IBD	HM
**DISEASED COLON (ULCERATIVE COLITIS)**						
1	Ochrobactrum	s__	0.1	Proteobacteria	Causes early bacterial dependent induction of inducible nitric oxide synthase (iNOS) in epithelial cells in experimental colitis	EU
2	Delftia	s__	0.1	Proteobacteria	Fecal and mucosa associated microbe in IBD	FM
3	Sphingomonas	s__	0.5	Proteobacteria	Tissue associated intestinal microflora	FF
4	Burkholderia	s__	0.2	Proteobacteria	Causes dysfunction of GALT and gut flora in IBD	FI
5	Acinetobacter	rhizosphaerae	0.7	Proteobacteria	Identified gut microbe in IBD	K
6	Acinetobacter	lwoffii	0.1	Proteobacteria	Gut bacteria in multiple sclerosis patients	W
7	Acinetobacter	s__	0.5	Proteobacteria	Tissue associated intestinal microflora	GF
8	Stenotrophomonas	geniculata	0.1	Proteobacteria	Identified gut microbe in IBD	AG
9	Enterococcus	s__	0.8	Firmicutes	Induces experimental IBD	CV
10	Staphylococcus	sciuri	0.1	Firmicutes	Develops intestinal inflammation in acute and chronic colitis	I
11	Staphylococcus	aureus	0.6	Firmicutes	Causes Crohn's disease	AU
12	Lactobacillus	zeae	1.9	Firmicutes	Maintains remission of ulcerative colitis	A
13	Lactobacillus	s__	0.4	Firmicutes	Maintains remission of ulcerative colitis	CW
14	Lactococcus	s__	0.7	Firmicutes	used in the treatment of Crohn's disease	CX
15	Pseudomonas	alcaligenes	1.8	Proteobacteria	Identified in the gut microbiota of IBD	AX
16	Pseudomonas	s__	0.1	Proteobacteria	Infection in Children with Early-onset Crohn's Disease	GG
17	Pseudomonas	Other	0.2	Proteobacteria	Gut microbe in children with early onset Crohn's disease	HR
18	Bacillus	s__	0.1	Firmicutes	Increases cytokine levels in IBD	CL
19	Bacteroides	caccae	0.1	Bacteroidetes	Identified in the gut of ulcerative colitis patients	AS
20	Bacteroides	Other	0.2	Bacteroidetes	Commensal bacteria that induces colitis	GR
21	Blautia	producta	0.1	Firmicutes	Gut microbe in Obesity and IBD	N
22	Faecalibacterium	prausnitzii	0.1	Firmicutes	Gut microbe in Crohn's disease patients	O
23	Microbacterium	maritypicum	0.1	Actinobacteria	Fecal microbiome in Obesity	V
24	Eggerthella	lenta	5.1	Actinobacteria	Causes bacteremia in Crohn's disease patient	AA
25	Propionibacterium	acnes	2.8	Actinobacteria	Intestinal microbe in Liver disease	BA
26	Methanobrevibacter	s__	0.3	Euryarchaeota	Identified in the gut of IBD	BB
27	g__	s__	1.8	Acidobacteria	Identified in the gut microbiome of Type 2 Diabetes patients	BD
28	g__	s__	0.3	Actinobacteria	Identified in gut microbiota in IBD	BF
29	Adlercreutzia	s__	0.2	Actinobacteria	Causes dysbiosis in IBD patients	BQ
30	Slackia	s__	0.2	Actinobacteria	Alters human gut microbiome in Multiple Sclerosis	BS
31	g__	s__	0.8	Bacteroidetes	Identified in gut microbiome of IBD patients	CA
32	g__	s__	0.4	Bacteroidetes	Characterized in intestinal biopsies in IBD patients	CB
33	g__	s__	0.1	Bacteroidetes	Human gut microbe in Obesity and IBD	CC
34	Cloacibacterium	s__	0.4	Bacteroidetes	Identified in the rectum of human colorectal adenoma patients	CG
35	g__	s__	0.1	Firmicutes	Causes microbiota dysbiosis in IBD	CO
36	g__	s__	0.1	Firmicutes	Gut microbe in IBD	DA
37	g__	s__	1.7	Firmicutes	A microbial signature of Crohn's disease	DB
38	Clostridium	s__	0.8	Firmicutes	Causes infection of the gut in IBD	DE
39	Dorea	s__	0.1	Firmicutes	Causes dysfunction of the intestinal microbiome in IBD	DJ
40	Lachnospira	s__	0.3	Firmicutes	gut bacteria in Crohn's disease patients	DK
41	Ruminococcus	s__	0.1	Firmicutes	Dominant in gut microbiome of IBD patients	DO
42	g__	s__	0.3	Firmicutes	A microbial signature of Crohn's disease	DQ
43	Oscillospira	s__	0.1	Firmicutes	Gut microbe in IBD patients	DU
44	Ruminococcus	s__	0.9	Firmicutes	Dominant in gut microbiome of IBD patients	DV
45	g__	s__	0.3	Firmicutes	Gut microbe underlying the onset of IBD	DW
46	Acidaminococcus	s__	1.1	Firmicutes	Gut microbe in IBD	DX
47	Phascolarctobacterium	s__	2.2	Firmicutes	Causes dysfunction of the intestinal microbiome in IBD	DZ
48	Schwartzia	s__	0.5	Firmicutes	Causes fecal microbial dysbiosis in IBD	EA
49	Anaerococcus	s__	0.6	Firmicutes	Microbe in Inflammatory Pouch Complications	EE
50	Finegoldia	s__	0.3	Firmicutes	Intestinal microbe in colorectal cancer	EF
51	g__	s__	0.6	Firmicutes	Gut microbe in GI diseases	EI
52	Bulleidia	s__	0.1	Firmicutes	Fecal-associated and mucosalassociated microbiota in irritable bowel syndrome patients	EJ
53	Coprobacillus	s__	0.1	Firmicutes	Alters Gut Microbiota in Psoriatic Arthritis	EL
54	Leptotrichia	s__	0.5	Fusobacteria	Causes gut mucosal inflammation in Rheumatoid arthritis patients	EO
55	g__	s__	3.6	Proteobacteria	Intestinal microbe in children with severe and complicated acute viral gastroenteritis	EV
56	g__	s__	0.5	Proteobacteria	Causes chronic inflammation in IBD	FB
57	g__	s__	0.1	Proteobacteria	Microbial factor associated with postoperative Crohn's disease	FD
58	g__	s__	1.5	Proteobacteria	Involved in host-microbial cross talk in IBD	FG
59	Sutterella	s__	0.1	Proteobacteria	Gut microbe in experimental colitis	FH
60	Lautropia	s__	0.2	Proteobacteria	Causes fecal microbial dysbiosis in IBD	FJ
61	g__	s__	0.5	Proteobacteria	Fecal and mucosa associated microbe in IBD	FK
62	Citrobacter	s__	0.3	Proteobacteria	Gut microbe in newly diagnosed with treatment-naïve Crohn's disease patients	FY
63	Halomonas	s__	0.8	Proteobacteria	Intestinal microflora in chronic kidney disease	GB
64	g__	s__	0.3	Proteobacteria	Bacteria in human Ulcerative Colitis patients	GH
65	Other	Other	0.2	Actinobacteria	Alters fecal microbiota in pediatric IBD patients	GO
66	Eggerthella	Other	0.1	Actinobacteria	Causes bacteremia in Crohn's disease patient	GQ
67	Other	Other	4.0	Firmicutes	Gut microbe in experimental colitis	GT
68	Other	Other	4.2	Firmicutes	Fecal and mucosa associated microbe in IBD	GW
69	Weissella	Other	1.2	Firmicutes	Gut microbe in IBD patients	GX
70	Other	Other	2.5	Proteobacteria	Causes microbial dysbiosis in pediatric Crohn's disease	HD
71	Other	Other	1.4	Proteobacteria	Fecal and mucosa associated microbe in IBD	HL
**DISEASED COLON (CROHN'S COLITIS)**						
1	Ochrobactrum	s__	0.1	Proteobacteria	Causes early bacterial dependent induction of inducible nitric oxide synthase (iNOS) in epithelial cells in experimental colitis	EU
2	Sphingomonas	s__	0.1	Proteobacteria	Tissue associated intestinal microflora	FF
3	Burkholderia	s__	1.6	Proteobacteria	Causes dysfunction of GALT and gut flora in IBD	FI
4	Acinetobacter	rhizosphaerae	1.8	Proteobacteria	Identified gut microbe in IBD	K
5	Acinetobacter	lwoffii	0.5	Proteobacteria	Gut bacteria in multiple sclerosis patients	W
6	Acinetobacter	Other	1.6	Proteobacteria	Tissue associated intestinal microflora in colitis patients	HQ
7	Stenotrophomonas	geniculata	0.3	Proteobacteria	Identified gut microbe in IBD	AG
8	Enterococcus	s__	0.1	Firmicutes	Induces experimental IBD	CV
9	Staphylococcus	sciuri	0.1	Firmicutes	Develops intestinal inflammation in acute and chronic colitis	I
10	Staphylococcus	aureus	0.6	Firmicutes	Causes Crohn's disease	AU
11	Lactobacillus	zeae	1.9	Firmicutes	Maintains remission of ulcerative colitis	A
12	Lactobacillus	s__	0.4	Firmicutes	Maintains remission of ulcerative colitis	CW
13	Lactococcus	s__	0.7	Firmicutes	Used in the treatment of Crohn's disease	CX
14	Pseudomonas	alcaligenes	1.8	Proteobacteria	Identified in the gut microbiota of IBD	AX
15	Pseudomonas	s__	0.1	Proteobacteria	Infection in Children with Early-onset Crohn's Disease	GG
16	Pseudomonas	Other	0.2	Proteobacteria	Gut microbe in children with early onset Crohn's disease	HR
17	Bacillus	thermoamylovorans	0.2	Firmicutes	A probiotic- normal flora of the gut	E
18	Bacillus	s__	2.6	Firmicutes	Increases cytokine levels in IBD	CL
19	Bacteroides	eggerthii	0.1	Bacteroidetes	Enhances colitis in mice	AJ
20	Bacteroides	Other	0.4	Bacteroidetes	Commensal bacteria that induces colitis	GR
21	Microbacterium	maritypicum	0.1	Actinobacteria	Fecal microbiome in Obesity	V
22	Eggerthella	lenta	4.5	Actinobacteria	Causes bacteremia in Crohn's disease patient	AA
23	Brevundimonas	diminuta	0.2	Proteobacteria	Identified in the adult fecal microbiota of allergy patients	AO
24	Propionibacterium	acnes	1.2	Actinobacteria	Intestinal microbe in Liver disease	BA
25	Methanobrevibacter	s__	0.8	Euryarchaeota	Identified in the gut of IBD patients	BB
26	g__	s__	0.7	Acidobacteria	Identified in the gut microbiome of Type 2 Diabetes patients	BD
27	g__	s__	0.2	Actinobacteria	Identified in gut microbiota in IBD	BE
28	g__	s__	0.5	Actinobacteria	Identified in gut microbiota in IBD	BF
29	Microbacterium	s__	0.1	Actinobacteria	Identified in the duodenum of children with ulcerative colitis	BK
30	Bifidobacterium	s__	0.2	Actinobacteria	Identified in gut microbiota of IBD patients	BO
31	g__	s__	0.3	Actinobacteria	Identified in fecal microbiota of pediatric IBD patients	BP
32	Atopobium	s__	0.2	Actinobacteria	Altered intestinal microbiota in Crohn's disease	BR
33	Slackia	s__	1.3	Actinobacteria	Alters human gut microbiome in Multiple Sclerosis	BS
34	g__	s__	0.1	Bacteroidetes	Identified in gut microbiome of IBD patients	CA
35	g__	s__	0.3	Bacteroidetes	Human gut microbe in Obesity and IBD	CC
36	g__	s__	0.7	Firmicutes	Causes microbiota dysbiosis in IBD	CO
37	g__	s__	0.2	Firmicutes	Gut microbe in IBD	DA
38	g__	s__	0.9	Firmicutes	A microbial signature of Crohn's disease	DB
39	Clostridium	s__	0.6	Firmicutes	Causes infection of the gut in IBD	DE
40	Lachnospira	s__	0.6	Firmicutes	Gut bacteria in Crohn's disease patients	DK
41	Moryella	s__	0.1	Firmicutes	Microbe in Inflammatory Pouch Complications	DL
42	g__	s__	0.1	Firmicutes	gut microbe in IBD	DP
43	g__	s__	0.6	Firmicutes	A microbial signature of Crohn's disease	DQ
44	Oscillospira	s__	0.2	Firmicutes	Gut microbe in IBD patients	DU
45	Ruminococcus	s__	0.9	Firmicutes	Dominant in gut microbiome of IBD patients	DV
46	g__	s__	0.2	Firmicutes	Gut microbe underlying the onset of IBD	DW
47	Acidaminococcus	s__	1.0	Firmicutes	Gut microbe in IBD	DX
48	Phascolarctobacterium	s__	0.6	Firmicutes	Causes dysfunction of the intestinal microbiome in IBD	DZ
49	Schwartzia	s__	0.2	Firmicutes	Causes fecal microbial dysbiosis in IBD	EA
50	g__	s__	0.1	Firmicutes	A microbial signature of Crohn's disease	EC
51	Anaerococcus	s__	0.3	Firmicutes	Microbe in Inflammatory Pouch Complications	EE
52	Finegoldia	s__	0.4	Firmicutes	Intestinal microbe in colorectal cancer	EF
53	g__	s__	0.5	Firmicutes	Gut microbe in GI diseases	EI
54	Bulleidia	s__	0.2	Firmicutes	Fecal-associated and mucosalassociated microbiota in irritable bowel syndrome patients	EJ
55	Coprobacillus	s__	0.3	Firmicutes	Alters Gut Microbiota in Psoriatic Arthritis	EL
56	Leptotrichia	s__	0.5	Fusobacteria	Causes gut mucosal inflammation in Rheumatoid arthritis patients	EO
57	g__	s__	4.1	Proteobacteria	Intestinal microbe in children with severe and complicated acute viral gastroenteritis	EV
58	g__	s__	0.2	Proteobacteria	Microbial factor associated with postoperative Crohn's disease	FD
59	g__	s__	1.2	Proteobacteria	Involved in host-microbial cross talk in IBD	FG
60	Sutterella	s__	0.1	Proteobacteria	Gut microbe in experimental colitis	FH
61	Lautropia	s__	0.1	Proteobacteria	Causes fecal microbial dysbiosis in IBD	FJ
62	g__	s__	0.4	Proteobacteria	Fecal and mucosa associated microbe in IBD	FK
63	g__	s__	0.1	Proteobacteria	Bacteria in Mucosal and Submucosal Intestinal Tissues in Advanced Crohn's Disease	FN
64	Ralstonia	s__	0.1	Proteobacteria	Microbiota in the Mucosa of Patients With Ulcerative Colitis	FP
65	Halomonas	s__	0.5	Proteobacteria	Intestinal microflora in chronic kidney disease	GB
66	Haemophilus	s__	0.7	Proteobacteria	Treatment naïve microbiome in new onset Crohn's disease	GD
67	g__	s__	0.1	Proteobacteria	Microbe in colon tissue from IBD subjects	GE
68	Other	Other	1.1	Actinobacteria	Alters fecal microbiota in pediatric IBD patients	GO
69	Eggerthella	Other	0.1	Actinobacteria	Causes bacteremia in Crohn's disease patient	GQ
70	Other	Other	1.7	Firmicutes	Gut microbe in experimental colitis	GT
71	Other	Other	2.2	Firmicutes	Fecal and mucosa associated microbe in IBD	GW
72	Weissella	Other	2.2	Firmicutes	Gut microbe in IBD patients	GX
73	Other	Other	0.1	Proteobacteria	Causes microbial dysbiosis in pediatric Crohn's disease	HD
74	Methylobacterium	Other	0.1	Proteobacteria	Causes gut microbial dysbiosis in pediatric Crohn's disease patients	HG
75	Other	Other	0.2	Proteobacteria	Fecal and mucosa associated microbe in IBD	HK
76	Other	Other	0.6	Proteobacteria	Fecal and mucosa associated microbe in IBD	HL

*Specific Information of functions was adapted from NCBI Genome Database (https://www.ncbi.nlm.nih.gov/genome/)*.

*The bacterial species that could not be identified at the genus level are mentioned as g___ and the bacterial species that could not be identified at the species level are mentioned as s___*.

### Statistical Analysis and Evaluation

Statistical analysis was performed between the healthy and diseased groups and based on race classification (*n* = 13 CC, *n* = 13 UC, *n* = 13 non-disease healthy patients, *n* = 30 CA and *n* = 9 AA). A non-parametric Mann-Whitney *U* Test *p*-value < 0.05 of bacterial 16S rRNA OTUs between the groups was considered statistically significant. IBM SPSS software package version 23 (IBM Analytics, USA) was used to conduct statistical analysis.

## Results

### Relative Abundance Analysis

**Figure 2:**
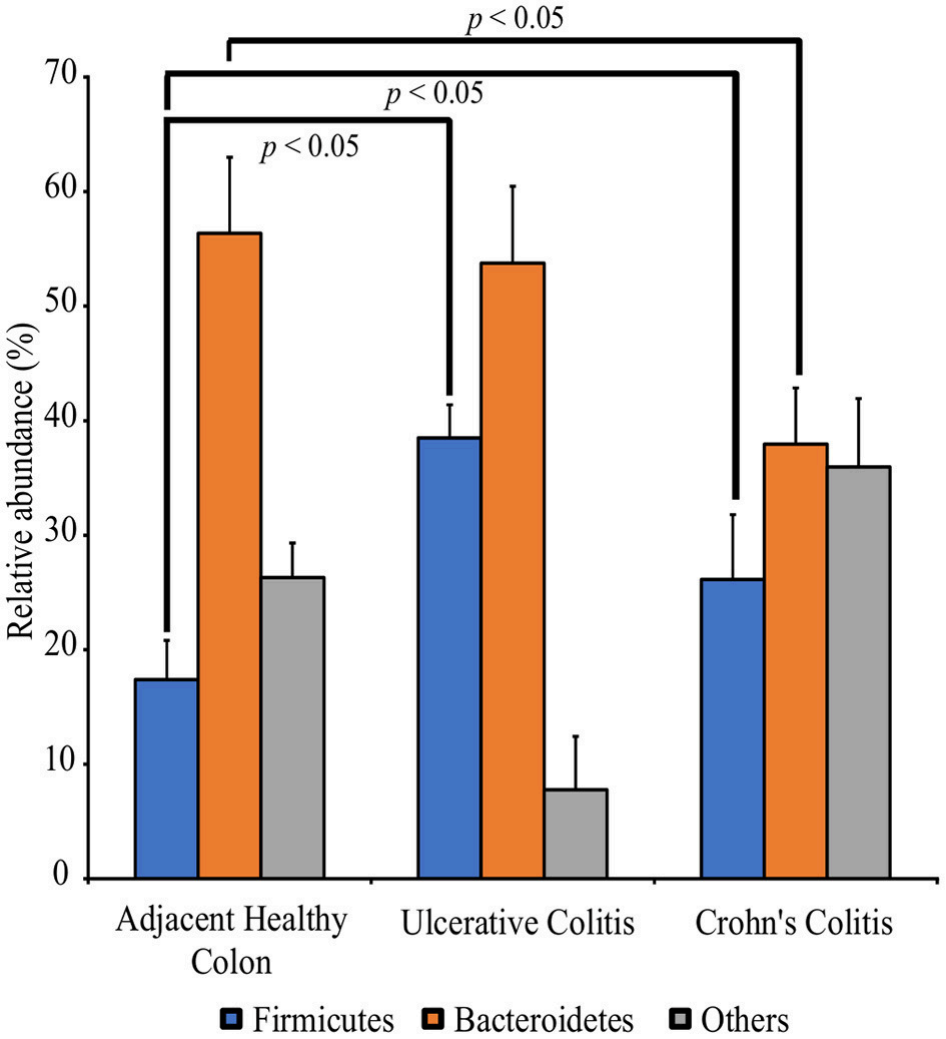
Perturbation of the full thickness colon microbiome in Colitis specimens as compared to adjacent healthy specimens. Specimens are categorized into adjacent healthy colon (*n* = 13), Ulcerative colitis (UC, *n* = 13) and Crohn's colitis (CC, *n* = 13). Data represented are the mean of relative abundances of each phylum identified in specimens belonging to each group while error bars indicate standard error. Diseased specimens demonstrate a balance between the Phyla Firmicutes and Bacteroidetes. Conversely, healthy colon specimens demonstrate a significantly higher proportion of Phyla Bacteroidetes. ^*^p < 0.05 by Mann-Whitney *U* Test (*n* = 13 under each group).

QIIME analysis showed about two hundred twenty-eight bacterial species in entire 39 specimens (Tables 1–**5**). However, non-ambiguous annotation at the species name resulted in fifty-three bacterial identifications. The dominant phyla across all samples (both diseased and healthy specimens) were Bacteroidetes (46.92%), followed by Firmicutes (27.8%), and Proteobacteria (24.5%). Most importantly, our results indicate that putative oral pathogens (belonging to mostly Phylum Firmicutes) dominated the microbiome of diseased specimens (Figure 1). Adjacent healthy specimens show an increased abundance of Phylum Bacteroidetes (~ 57%, containing mostly symbiotic and/or beneficial bacteria) population, which is altered in disease categories (Figure 2).

### Differential Expression of Microbiomes in the Colon of CA and AA Patients

**Figure 3:**
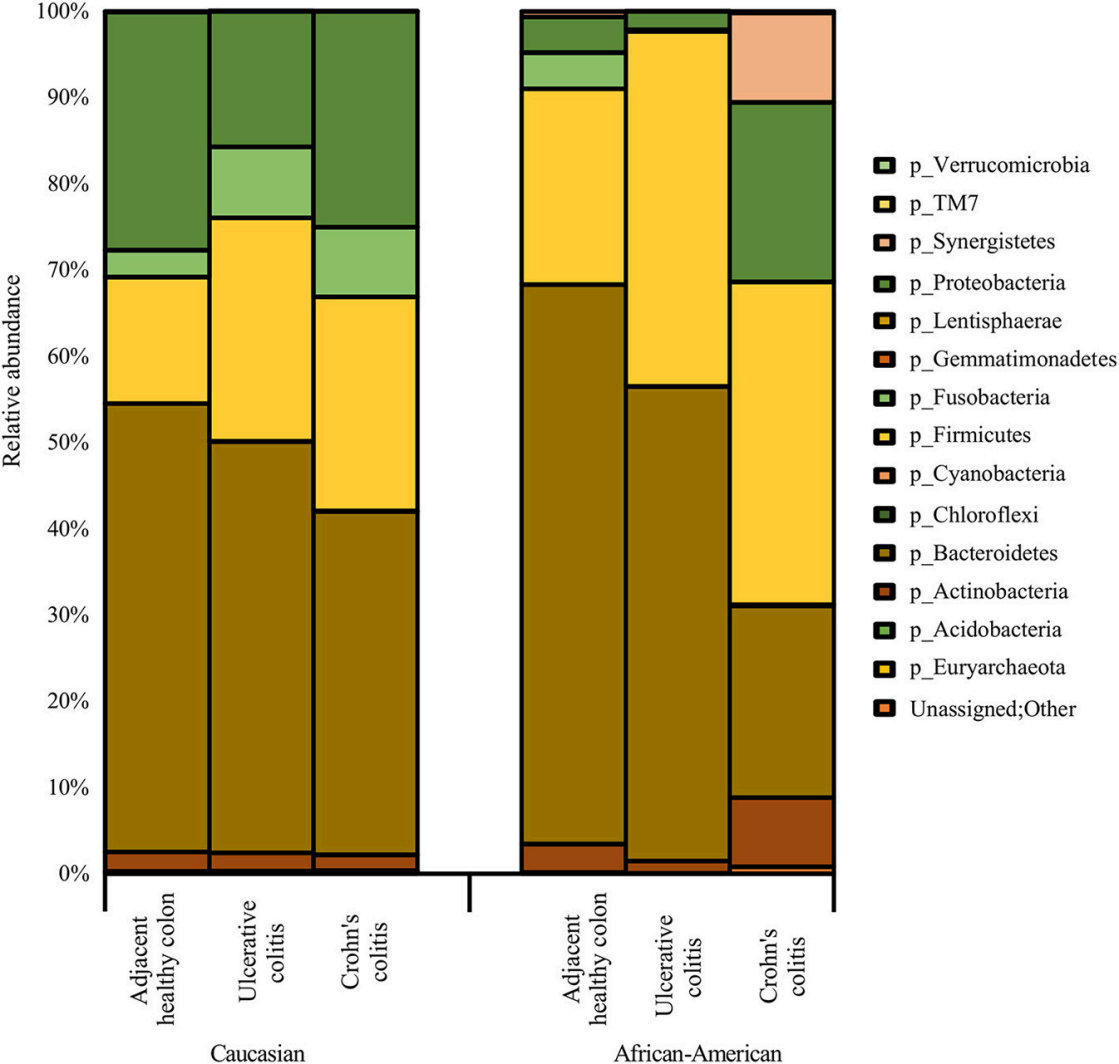
Summary of major bacterial taxa showing the relative abundance of oral and gut bacteria at the Phylum level in the colitis and adjacent healthy specimen groups under each race. Data represented are the mean of relative abundances of each Phyla detected in samples belonging to each group. The dominant phyla across all samples (both diseased and healthy specimens) were Bacteroidetes, followed by Firmicutes and Proteobacteria. Other major phyla observed among these specimens also include Actinobacteria, Fusobacteria, and Synergistetes. The Phylum Proteobacteria did not show any significant difference between healthy colon specimens and diseased colon specimens. A larger proportion of unassigned bacteria (0.3%) was identified in AA Crohn's Colitis patients compared to other groups.

Figure 3 show racial differences of various bacterial phyla in adjacent healthy, UC and CC full thickness colon specimens. The tissue specimens from Caucasians represented a significantly higher proportion (*p* < 0.05) of the oral pathogen, *Fusobacterium*, and gut bacteria, *Parabacteroides* (Bacteroidetes). CA specimens also showed significantly higher levels (*p* < 0.05) of Phyla Proteobacteria including *Citrobacter, Hemophilus, Acinetobacter, Pseudomonas*, and *Stenotrophomonas* as compared to AA. Whereas, the AA specimens were observed to have a significantly higher proportion (*p* < 0.05) of *Prevotella* (Bacteroidetes) and *Clostridia* (Firmicutes) (Figure 3; Table 4).

**Table 4:** Functions and Proportions of bacterial species identified in the full thickness human colon specimens of Caucasians and African Americans.

**Sl. No**.	**Bacteria genus**	**Bacteria species**	**Proportion (%)**	**Bacteria phylum**	**Function in IBD**	**NCBI genome database link**
**CAUCASIAN AMERICANS**						
1	Lactobacillus	zeae	6.8	Firmicutes	Maintains remission of ulcerative colitis	A
2	Bacillus	thermoamylovorans	0.1	Firmicutes	A probiotic- normal flora of the gut	E
3	Prevotella	tannerae	0.1	Bacteroidetes	Prevalent in colitis	F
4	Collinsella	stercoris	0.0	Actinobacteria	Used for treatment of IBD	G
5	Prevotella	stercorea	1.4	Bacteroidetes	Alters mucosal microbiota in the colon of patients with IBD	H
6	Staphylococcus	sciuri	0.1	Firmicutes	Develops intestinal inflammation in acute and chronic colitis	I
7	Shuttleworthia	satelles	0.0	Firmicutes	Identified in the human ileum	J
8	Acinetobacter	rhizosphaerae	1.1	Proteobacteria	Identified gut microbe in IBD	K
9	Blautia	producta	0.1	Firmicutes	Gut microbe in Obesity and IBD	N
10	Akkermansia	muciniphila	0.1	Verrucomicrobia	Adheres to enterocytes and strengthens the integrity of the epithelial cell layer	S
11	Prevotella	melaninogenica	0.2	Bacteroidetes	Gut microbiome biomarker in ankylosing spondylitis	U
12	Acinetobacter	lwoffii	0.2	Proteobacteria	Gut bacteria in multiple sclerosis patients	W
13	Bifidobacterium	longum	0.2	Actinobacteria	Attenuates acute murine experimental model of IBD	Y
14	Eggerthella	lenta	4.3	Actinobacteria	Causes bacteremia in Crohn's disease patient	AA
15	Rhizobium	leguminosarum	0.1	Proteobacteria	Identified gut microbe in IBD patients	AB
16	Anoxybacillus	kestanbolensis	0.1	Firmicutes	Identified gut microbe in IBD patients	AD
17	Stenotrophomonas	geniculata	0.1	Proteobacteria	Identified gut microbe in IBD patients	AG
18	Corynebacterium	durum	0.1	Actinobacteria	Identified gut microbe in IBD patients	AK
19	Eubacterium	dolichum	0.8	Firmicutes	Causes dysbiosis of the intestinal microbiota	AL
20	Brevundimonas	diminuta	0.1	Proteobacteria	Identified in the adult fecal microbiota of allergy patients	AO
21	Lysinibacillus	boronitolerans	12.3	Firmicutes	Identified gut microbe in IBD patients	AT
22	Staphylococcus	aureus	0.6	Firmicutes	Causes Crohn's disease	AU
23	Peptostreptococcus	anaerobius	4.8	Firmicutes	Causes dysbiosis in IBD	AW
24	Pseudomonas	alcaligenes	1.0	Proteobacteria	Identified in the gut microbiota of IBD	AX
25	Propionibacterium	acnes	3.9	Actinobacteria	Intestinal microbe in Liver disease	BA
26	Methanobrevibacter	s__	0.6	Euryarchaeota	Identified in the gut of IBD	BB
27	g__	s__	0.8	Acidobacteria	Identified in the gut microbiome of Type 2 Diabetes patients	BD
28	g__	s__	0.9	Actinobacteria	Identified in gut microbiota in IBD	BF
29	Varibaculum	s__	0.1	Actinobacteria	Identified in the gut of a premature infant	BH
30	Corynebacterium	s__	0.7	Actinobacteria	Causes experimental colitis	BI
31	Microbacterium	s__	0.1	Actinobacteria	Identified in the duodenum of children with ulcerative colitis	BK
32	Bifidobacterium	s__	0.1	Actinobacteria	identified in gut microbiota of IBD patients	BO
33	g__	s__	0.2	Actinobacteria	Identified in fecal microbiota of pediatric IBD patients	BP
34	Adlercreutzia	s__	0.1	Actinobacteria	Causes dysbiosis in IBD patients	BQ
35	Atopobium	s__	0.1	Actinobacteria	altered intestinal microbiota in Crohn's disease	BR
36	Slackia	s__	0.7	Actinobacteria	Alters human gut microbiome in Multiple Sclerosis	BS
37	Prevotella	s__	0.1	Bacteroidetes	A microbial signature of Crohn's disease	BZ
38	g__	s__	0.4	Bacteroidetes	Identified in gut microbiome of IBD patients	CA
39	g__	s__	0.2	Bacteroidetes	Characterized in intestinal biopsies in IBD patients	CB
40	g__	s__	0.2	Bacteroidetes	Human gut microbe in Obesity and IBD	CC
41	Cloacibacterium	s__	0.7	Bacteroidetes	Identified in the rectum of human colorectal adenoma patients	CG
42	SHD-231	s__	0.1	Chloroflexi	Identified in the fecal microbiome of Gout patients	CH
43	g__	s__	0.1	Cyanobacteria	Identified in the gut microbiome of IBD patients	CI
44	Calothrix	s__	0.1	Cyanobacteria	Identified gut microbe in IBD patients	CK
45	Bacillus	s__	0.5	Firmicutes	Increases cytokine levels in IBD	CL
46	g__	s__	0.6	Firmicutes	Causes microbiota dysbiosis in IBD	CO
47	Gemella	s__	0.1	Firmicutes	Microbiome in New-Onset Crohn's Disease	CP
48	Abiotrophia	s__	0.1	Firmicutes	Causes fecal microbial dysbiosis in IBD	CS
49	Alloiococcus	s__	3.4	Firmicutes	Identified gut microbe in IBD patients	CT
50	Enterococcus	s__	0.4	Firmicutes	Induces experimental IBD	CV
51	Lactobacillus	s__	0.5	Firmicutes	Maintains remission of ulcerative colitis	CW
52	Lactococcus	s__	0.7	Firmicutes	Used in the treatment of Crohn's disease	CX
53	g__	s__	0.1	Firmicutes	Gut microbe in IBD	DA
54	g__	s__	1.1	Firmicutes	A microbial signature of Crohn's disease	DB
55	Christensenella	s__	1.2	Firmicutes	Identified gut microbe	DC
56	Clostridium	s__	0.9	Firmicutes	causes infection of the gut in IBD	DE
57	Pseudoramibacter_Eubacterium	s__	1.8	Firmicutes	Metabolizes Linoleic acid in the Gut	DF
58	g__	s__	0.1	Firmicutes	Commensal gut bacteria in IBD	DG
59	Blautia	s__	0.3	Firmicutes	Butyrate-producing bacterial species in Gut	DH
60	Coprococcus	s__	0.2	Firmicutes	Butyrate-producing bacterial species in Gut	DI
61	Dorea	s__	0.1	Firmicutes	Causes dysfunction of the intestinal microbiome in IBD	DJ
62	Lachnospira	s__	0.3	Firmicutes	Gut bacteria in Crohn's disease patients	DK
63	g__	s__	0.1	Firmicutes	Gut microbe in IBD	DP
64	g__	s__	0.6	Firmicutes	A microbial signature of Crohn's disease	DQ
65	Peptostreptococcus	s__	0.3	Firmicutes	Causes gut microbiota dysbiosis in IBD	DR
66	Anaerotruncus	s__	0.1	Firmicutes	Tissue associated intestinal microflora	DT
67	Oscillospira	s__	0.2	Firmicutes	Gut microbe in IBD patients	DU
68	Ruminococcus	s__	0.4	Firmicutes	Dominant in gut microbiome of IBD patients	DV
69	g__	s__	0.3	Firmicutes	Gut microbe underlying the onset of IBD	DW
70	Acidaminococcus	s__	1.2	Firmicutes	Gut microbe in IBD	DX
71	Phascolarctobacterium	s__	1.9	Firmicutes	Causes dysfunction of the intestinal microbiome in IBD	DZ
72	Schwartzia	s__	0.5	Firmicutes	Causes fecal microbial dysbiosis in IBD	EA
73	Selenomonas	s__	0.2	Firmicutes	Causes dysbiosis in colorectal cancer	EB
74	g__	s__	0.1	Firmicutes	A microbial signature of Crohn's disease	EC
75	Anaerococcus	s__	0.7	Firmicutes	Microbe in Inflammatory Pouch Complications	EE
76	Finegoldia	s__	0.3	Firmicutes	Intestinal microbe in colorectal cancer	EF
77	g__	s__	0.4	Firmicutes	Gut microbe in GI diseases	EI
78	Bulleidia	s__	0.1	Firmicutes	Fecal-associated and mucosalassociated microbiota in irritable bowel syndrome patients	EJ
79	Coprobacillus	s__	0.3	Firmicutes	Alters Gut Microbiota in Psoriatic Arthritis	EL
80	Fusobacterium	s__	0.8	Fusobacteria	Identified from colonic biopsies of IBD patients	EN
81	Leptotrichia	s__	0.2	Fusobacteria	Causes gut mucosal inflammation in Rheumatoid arthritis patients	EO
82	g__	s__	0.3	Gemmatimonadetes	Identified gut microbe in IBD patients	EP
83	g__	s__	0.4	Lentisphaerae	Normal gut microbe	EQ
84	Ochrobactrum	s__	0.1	Proteobacteria	Causes early bacterial dependent induction of inducible nitric oxide synthase (iNOS) in epithelial cells in experimental colitis	EU
85	g__	s__	4.7	Proteobacteria	Intestinal microbe in children with severe and complicated acute viral gastroenteritis	EV
86	g__	s__	0.2	Proteobacteria	Causes chronic inflammation in IBD	FB
87	g__	s__	0.3	Proteobacteria	Identified gut microbe in IBD patients	FC
88	g__	s__	0.1	Proteobacteria	Microbial factor associated with postoperative Crohn's disease	FD
89	Sphingomonas	s__	0.3	Proteobacteria	Tissue associated intestinal microflora	FF
90	g__	s__	1.7	Proteobacteria	Involved in host-microbial cross talk in IBD	FG
91	Sutterella	s__	0.1	Proteobacteria	gut microbe in experimental colitis	FH
92	Burkholderia	s__	1.2	Proteobacteria	Causes dysfunction of GALT and gut flora in IBD	FI
93	Lautropia	s__	0.2	Proteobacteria	Causes fecal microbial dysbiosis in IBD	FJ
94	g__	s__	0.4	Proteobacteria	Fecal and mucosa associated microbe in IBD	FK
95	Comamonas	s__	2.5	Proteobacteria	Identified gut microbe in IBD patients	FL
96	Delftia	s__	0.1	Proteobacteria	Fecal and mucosa associated microbe in IBD	FM
97	Desulfovibrio	s__	0.2	Proteobacteria	Sulfate reducing bacteria in IBD	FV
98	Citrobacter	s__	0.2	Proteobacteria	Gut microbe in newly diagnosed with treatment-naïve Crohn's disease patients	FY
99	Halomonas	s__	0.9	Proteobacteria	Intestinal microflora in chronic kidney disease	GB
100	Aggregatibacter	s__	0.6	Proteobacteria	Causes fungal microbiota dysbiosis in IBD	GC
101	Haemophilus	s__	0.3	Proteobacteria	Treatment naïve microbiome in new onset Crohn's disease	GD
102	Pseudomonas	s__	0.8	Proteobacteria	Infection in Children with Early-onset Crohn's Disease	GG
103	g__	s__	0.2	Proteobacteria	Bacteria in human Ulcerative Colitis patients	GH
104	g__	s__	0.1	TM7	No role in IBD
105	Other	Other	0.5	Actinobacteria	Alters fecal microbiota in pediatric IBD patients	GO
106	Eggerthella	Other	0.1	Actinobacteria	Causes bacteremia in Crohn's disease patient	GQ
107	Bacteroides	Other	0.1	Bacteroidetes	Commensal bacteria that induces colitis	GR
108	Prevotella	Other	0.7	Bacteroidetes	A microbial signature of Crohn's disease	GS
109	Other	Other	3.2	Firmicutes	Gut microbe in experimental colitis	GT
110	Other	Other	7.5	Firmicutes	Fecal and mucosa associated microbe in IBD	GW
111	Weissella	Other	1.5	Firmicutes	Gut microbe in IBD patients	GX
112	Other	Other	0.1	Firmicutes	Commensal gut bacteria in IBD	HA
113	Other	Other	1.1	Proteobacteria	Causes microbial dysbiosis in pediatric Crohn's disease	HD
114	Paracoccus	Other	0.3	Proteobacteria	Identified gut microbe in IBD patients	HH
115	Other	Other	0.1	Proteobacteria	Fecal and mucosa associated microbe in IBD	HK
116	Other	Other	0.7	Proteobacteria	Fecal and mucosa associated microbe in IBD	HL
117	Other	Other	0.1	Proteobacteria	Identified gut microbe in IBD patients	HN
118	Other	Other	0.5	Proteobacteria	Adult fecal microbe	HO
**AFRICAN AMERICANS**						
1	Lactobacillus	zeae	1.1	Firmicutes	Maintains remission of ulcerative colitis	A
2	Prevotella	stercorea	4.6	Bacteroidetes	Alters mucosal microbiota in the colon of patients with IBD	H
3	Shuttleworthia	satelles	0.4	Firmicutes	Identified in the human ileum	J
4	Acinetobacter	rhizosphaerae	0.2	Proteobacteria	Identified gut microbe in IBD patients	K
5	Microbacterium	maritypicum	0.2	Actinobacteria	Fecal microbiome in Obesity	V
6	Acinetobacter	lwoffii	0.5	Proteobacteria	Gut bacteria in multiple sclerosis patients	W
7	Eggerthella	lenta	0.7	Actinobacteria	Causes bacteremia in Crohn's disease patient.	AA
8	Stenotrophomonas	geniculata	2.0	Proteobacteria	Identified gut microbe in IBD patients	AG
9	Corynebacterium	durum	0.1	Actinobacteria	Identified gut microbe in IBD patients	AK
10	Eubacterium	dolichum	0.5	Firmicutes	Causes dysbiosis of the intestinal microbiota	AL
11	Brevundimonas	diminuta	0.1	Proteobacteria	Identified in the adult fecal microbiota of allergy patients	AO
12	Lysinibacillus	boronitolerans	5.0	Firmicutes	Identified gut microbe in IBD patients	AT
13	Staphylococcus	aureus	0.2	Firmicutes	Causes Crohn's disease	AU
14	Peptostreptococcus	anaerobius	24.9	Firmicutes	Causes dysbiosis in IBD	AW
15	Pseudomonas	alcaligenes	1.1	Proteobacteria	Identified in the gut microbiota of IBD	AX
16	Propionibacterium	acnes	0.6	Actinobacteria	Intestinal microbe in Liver disease	BA
17	Methanobrevibacter	s__	0.4	Euryarchaeota	Identified in the gut of IBD	BB
18	g__	s__	0.2	Acidobacteria	Identified in human gut microbiota	BC
19	g__	s__	4.3	Acidobacteria	Identified in the gut microbiome of Type 2 Diabetes patients	BD
20	g__	s__	0.3	Actinobacteria	Identified in gut microbiota in IBD	BE
21	Varibaculum	s__	0.6	Actinobacteria	Identified in the gut of a premature infant	BH
22	Corynebacterium	s__	0.5	Actinobacteria	Causes experimental colitis	BI
23	Arthrobacter	s__	0.2	Actinobacteria	Fecal microflora in chronic IBD patients	BL
24	Slackia	s__	0.2	Actinobacteria	Alters human gut microbiome in Multiple Sclerosis	BS
25	Chryseobacterium	s__	0.3	Bacteroidetes	Fecal and mucosa associated microbe in IBD	CF
26	Cloacibacterium	s__	0.2	Bacteroidetes	Identified in the rectum of human colorectal adenoma patients	CG
27	g__	s__	0.2	Cyanobacteria	Identified in human gut microbiota	CJ
28	Bacillus	s__	2.7	Firmicutes	Increases cytokine levels in IBD	CL
29	Alloiococcus	s__	6.0	Firmicutes	Identified gut microbe in IBD patients	CT
30	Lactobacillus	s__	0.2	Firmicutes	Maintains remission of ulcerative colitis	CW
31	Lactococcus	s__	0.3	Firmicutes	Used in the treatment of Crohn's disease	CX
32	g__	s__	0.1	Firmicutes	Gut microbe in IBD	DA
33	g__	s__	1.3	Firmicutes	A microbial signature of Crohn's disease	DB
34	Christensenella	s__	0.2	Firmicutes	Identified gut microbe	DC
35	Clostridium	s__	0.4	Firmicutes	causes infection of the gut in IBD	DE
36	Pseudoramibacter_Eubacterium	s__	0.9	Firmicutes	Metabolizes Linoleic acid in the Gut	DF
37	Lachnospira	s__	0.3	Firmicutes	Gut bacteria in Crohn's disease patients	DK
38	g__	s__	0.3	Firmicutes	A microbial signature of Crohn's disease	DQ
39	Peptostreptococcus	s__	0.4	Firmicutes	Causes gut microbiota dysbiosis in IBD	DR
40	Oscillospira	s__	0.2	Firmicutes	Gut microbe in IBD patients	DU
41	Ruminococcus	s__	2.2	Firmicutes	Dominant in gut microbiome of IBD patients	DV
42	g__	s__	0.4	Firmicutes	Gut microbe underlying the onset of IBD	DW
43	Acidaminococcus	s__	1.0	Firmicutes	Gut microbe in IBD	DX
44	Phascolarctobacterium	s__	0.5	Firmicutes	Causes dysfunction of the intestinal microbiome in IBD	DZ
45	Schwartzia	s__	0.1	Firmicutes	Causes fecal microbial dysbiosis in IBD	EA
46	Selenomonas	s__	0.5	Firmicutes	Causes dysbiosis in colorectal cancer	EB
47	Anaerococcus	s__	0.5	Firmicutes	Microbe in Inflammatory Pouch Complications	EE
48	Finegoldia	s__	0.4	Firmicutes	Intestinal microbe in colorectal cancer	EF
49	g__	s__	0.8	Firmicutes	Gut microbe in GI diseases	EI
50	Bulleidia	s__	0.1	Firmicutes	Fecal-associated and mucosalassociated microbiota in irritable bowel syndrome patients	EJ
51	Fusobacterium	s__	8.4	Fusobacteria	Identified from colonic biopsies of IBD patients	EN
52	Leptotrichia	s__	1.7	Fusobacteria	Causes gut mucosal inflammation in Rheumatoid arthritis patients	EO
53	g__	s__	0.7	Gemmatimonadetes	Identified gut microbe in IBD patients	EP
54	Ochrobactrum	s__	0.1	Proteobacteria	Causes early bacterial dependent induction of inducible nitric oxide synthase (iNOS) in epithelial cells in experimental colitis	EU
55	g__	s__	1.5	Proteobacteria	Intestinal microbe in children with severe and complicated acute viral gastroenteritis	EV
56	g__	s__	0.1	Proteobacteria	Involved in host-microbial cross talk in IBD	FG
57	Sutterella	s__	0.2	Proteobacteria	Gut microbe in experimental colitis	FH
58	Burkholderia	s__	0.4	Proteobacteria	Causes dysfunction of GALT and gut flora in IBD	FI
59	Lautropia	s__	0.2	Proteobacteria	Causes fecal microbial dysbiosis in IBD	FJ
60	Comamonas	s__	0.4	Proteobacteria	Identified gut microbe in IBD patients	FL
61	Ralstonia	s__	0.2	Proteobacteria	Microbiota in the Mucosa of Patients With Ulcerative Colitis	FP
62	Bilophila	s__	0.1	Proteobacteria	Causes irritable bowel syndrome	FU
63	Desulfovibrio	s__	0.1	Proteobacteria	Sulfate reducing bacteria in IBD	FV
64	Halomonas	s__	0.7	Proteobacteria	Intestinal microflora in chronic kidney disease	GB
65	g__	s__	0.1	Proteobacteria	Microbe in colon tissue from IBD subjects	GE
66	Acinetobacter	s__	0.7	Proteobacteria	Tissue associated intestinal microflora	GF
67	Pseudomonas	s__	0.1	Proteobacteria	Infection in Children with Early-onset Crohn's Disease	GG
68	g__	s__	0.8	TM7	No role in IBD
69	Other	Other	0.3	Actinobacteria	Alters fecal microbiota in pediatric IBD patients	GO
70	Eggerthella	Other	0.1	Actinobacteria	Causes bacteremia in Crohn's disease patient	GQ
71	Bacteroides	Other	0.7	Bacteroidetes	Commensal bacteria that induces colitis	GR
72	Prevotella	Other	3.2	Bacteroidetes	A microbial signature of Crohn's disease	GS
73	Other	Other	2.8	Firmicutes	Gut microbe in experimental colitis	GT
74	Paenibacillus	Other	0.1	Firmicutes	Gut microbe in a healthy infant	GU
75	Other	Other	2.2	Firmicutes	Fecal and mucosa associated microbe in IBD	GW
76	Paracoccus	Other	0.1	Proteobacteria	Identified gut microbe in IBD patients	HH
77	Other	Other	0.6	Proteobacteria	Fecal and mucosa associated microbe in IBD	HL
78	Other	Other	0.1	Proteobacteria	Adult fecal microbe	HO
79	Other	Other	0.4	Proteobacteria	Mucosal and fecal microbe	HP
80	Acinetobacter	Other	2.2	Proteobacteria	Tissue associated intestinal microflora in colitis patients	HQ
81	Pseudomonas	Other	0.2	Proteobacteria	Gut microbe in children with early onset Crohn's disease	HR

*Specific Information of functions was adapted from NCBI Genome Database (https://www.ncbi.nlm.nih.gov/genome/)*.

*The bacterial species that could not be identified at the genus level are mentioned as g___ and the bacterial species that could not be identified at the species level are mentioned as s___*.

As depicted in Figure 3, the adjacent healthy colon specimens, UC and CC contained ~ 1%, ~ 7% and ~ 7% of sequence reads, respectively that were un-assignable to any taxon with a larger proportion of them identified in AA Colitis patients. Other major phyla observed among these specimens also include Proteobacteria (Adjacent healthy: 23.8%; UC: 26.5% and CC: 23.1%), Actinobacteria (Adjacent healthy: 6.7%; UC: 8.1% and CC: 14.1%), Fusobacteria (Adjacent healthy: 4.2%; UC: 3.6% and CC: 4.0%), and Synergistetes (Adjacent healthy: 0.2%; UC: 0.04% and CC: 1.5%). The Phylum Proteobacteria did not show any significant difference between healthy colon specimens and diseased colon specimens (Table 4).

### Bacterial Species Identified in a Significantly Higher Proportion in Diseased Colon Tissues

As shown in Figure 3, diseased colon specimens represented a significantly higher proportion (*p* < 0.05) of gut bacteria belonging to Phylum Firmicutes including *Blautia producta, Faecalibacterium prausnitzii, Anoxybacillus kestanbolensis, Ruminococcus gnavus, Eubacterium dolichum, Lysinibacillus boronitolerans*, and oral bacteria including *Staphylococcus sciuri, Staphylococcus aureus, Streptococcus anginosus*.

In contrast, healthy colon specimens were significantly dominated (*p* < 0.05) by oral bacteria belonging to Phylum Actinobacteria that includes; *Corynebacterium kroppenstedtii, Corynebacterium durum*. Additionally, healthy colon specimens were dominated by gut bacteria belonging to Phylum Actinobacteria that includes; *Colinsella stercoris, Colinsella aerofaciens, Kocuria rhizophila, Eggerthella lenta, Propionibacterium granulosum, Propionibacterium acnes, Actinomyces europaeus, Rothia dentocariosa*, and Phylum Bacteroidetes that includes; *Bacteroides fragilis, Bacteroides eggerthii, Bacteroides caccae, Parabacteroides distasonis* (Figure 3).

### Alpha Diversity and Beta Diversity Analyses

Alpha diversity and beta diversity metrics were computed to analyse the diversity of bacterial species within each sample and between samples. To assess our sampling efficiency, we plotted rarefaction curves (Chao1 and Shannon) for all 39 specimens. Increased diversity (Shannon) in the diseased samples compared to control samples was observed. From the rarefaction curves, it is evident that most AA samples require additional sampling whereas Caucasian samples do not (data not shown).

Since, outliers exhibiting different microbiome profiles were observed both in the healthy and disease groups, we performed principle coordinate analysis (PCoA analysis) and hierarchial clustering to obtain a holistic view of the microbiome profile in each sample. Two dimensional PCoA plots revealed that control samples which had similar microbiome profiles as suggested by histograms and OTU heat map clustered together (data not shown).

### Pathogenic Oral and Gut Flora Abundantly Colonized in Diseased Colon Specimens

The pathogenic oral bacteria identified abundantly in diseased colon specimens as compared to healthy colon specimens were *Porphyromonas, Prevotella, Gemella, Staphylococcus, Streptococcus, Abiotrophia, Granulicatella, Lactobacillus, Lactococcus, Peptostreptococcus, Selenomonas, Veillonella, Parvimonas, Eubacterium, Fusobacterium, Pseudomonas, Aggregatibacter*, and *Corynebacterium* (Table 1).

Pathogenic gut bacteria identified abundantly in diseased colon specimens as compared to healthy colon specimens include *Ochrobactrum, Delftia, Sphingomonas, Burkholderia, Acinetobacter, Stenotrophomonas, Enterococcus, Granulicatella, Staphylococcus, Streptococcus, Lactobacillus, Lactococcus, Pseudomonas, Bacillus, Campylobacter*, and *Bacteroides* (Table 3).

## Discussion

Our study demonstrates significant perturbations among bacteria belonging to Phyla Bacteroidetes and Firmicutes in full-thickness diseased colon specimens containing neuromuscular compartment (Figure 2). Our studies further show that the proportion of pathogenic bacteria are higher in diseased compared to adjacent healthy colon specimens. We suggest that pathogenic bacteria belonging to these two phyla have a greater impact on colon motility function in colitis patients (Tables 1, 3). Although the incidence of IBD is increasing among African Americans (AA), the underlying causes are completely unknown (Sofia et al., 2014). Our study further highlight a significant disparity in bacterial dysbiosis among AA compared to CA colitis patients (Figure 3).

CA specimens had significantly higher levels of *Fusobacterium, Parabacteroides, Citrobacter, Haemophilus, Acinetobacter, Pseudomonas*, and *Stenotrophomonas*. *Fusobacterium nucleatum* is known to have a well-characterized role in the oral cavity. We have determined that *Fusobacterium* can be recovered from human full thickness colon specimens and this could indicate their ability to survive and proliferate inside host cells. *Parabacteroides* was found to be dominant in the acute phase of IBD in CA patients. *Citrobacter* is an epithelial cell adherent pathogen and can subvert inflammation in colitis. *Pseudomonas* interacts with the mucosal layer of colon and disrupts the mucosal barrier integrity leading to colitis in CA patients.

The AA specimens had significantly higher levels of *Prevotella* and *Clostridia*. *Prevotella* augments T-helper cells mediated colon mucosal inflammation by activating Toll-like receptor 2 leading to production of T-helper cells polarizing cytokines by antigen-presenting cells, including interleukins. In addition, *Prevotella* induce epithelial cells to produce interleukins and cytokines that can promote recruitment of neutrophils and mucosal T-helper cell immune responses. *Prevotella* can mediate inflammation of the mucosa leading to the circulation of bacteria, bacterial products and other inflammatory mediators. *Prevotella* could augment release of inflammatory mediators from immune cells and various stromal cells in colitis in AA patients. *Clostridium* can disrupt gut immune dormancy and cause infectious colitis in AA patients. Collectively, our data suggest that the presence of pathogenic bacteria in AA full thickness diseased specimens could adversely affect colon motility.

Additionally, our data in UC and CC specimens show the presence of several orange (*Prevotella, Peptostreptococcus, Eubacterium, Fusobacterium*, and *Campylobacter*), red (*Porphyromonas*), purple (*Veillonella*), and yellow (*Streptococcus*) complex putative oral pathogens known to cause gingivitis and periodontitis among IBD patients (Tables 1, 3). Previous studies using mucosal biopsies and feces have shown that gut microbiota in bowel diseases is characterized by an increase in certain phyla such as Proteobacteria, Firmicutes, genus *Bifidobacterium*, as well as a reduction in the amounts of genera *Ruminococcus, Clostridia* and (in some cases) *Faecalibacterium* (Lane et al., 2017; Nishida et al., 2018). However, none of the earlier studies using feces have shown a shift in the balance between Phyla Bacteroidetes and Firmicutes among UC or CC patients; even though this was observed in healthy individuals (Mariat et al., 2009; Koliada et al., 2017). In contrary to our results, one study using mucosal biopsies has shown a significantly decreased Firmicutes to Bacteroidetes ratio in both UC and CC compared with controls (Kabeerdoss et al., 2015). Collectively, our data suggest that the putative oral pathogens found in diseased colon specimens may modulate the proportion of non-detrimental gut bacteria, thus potentially worsening the condition of the colon in colitis patients.

**Figure 4:**
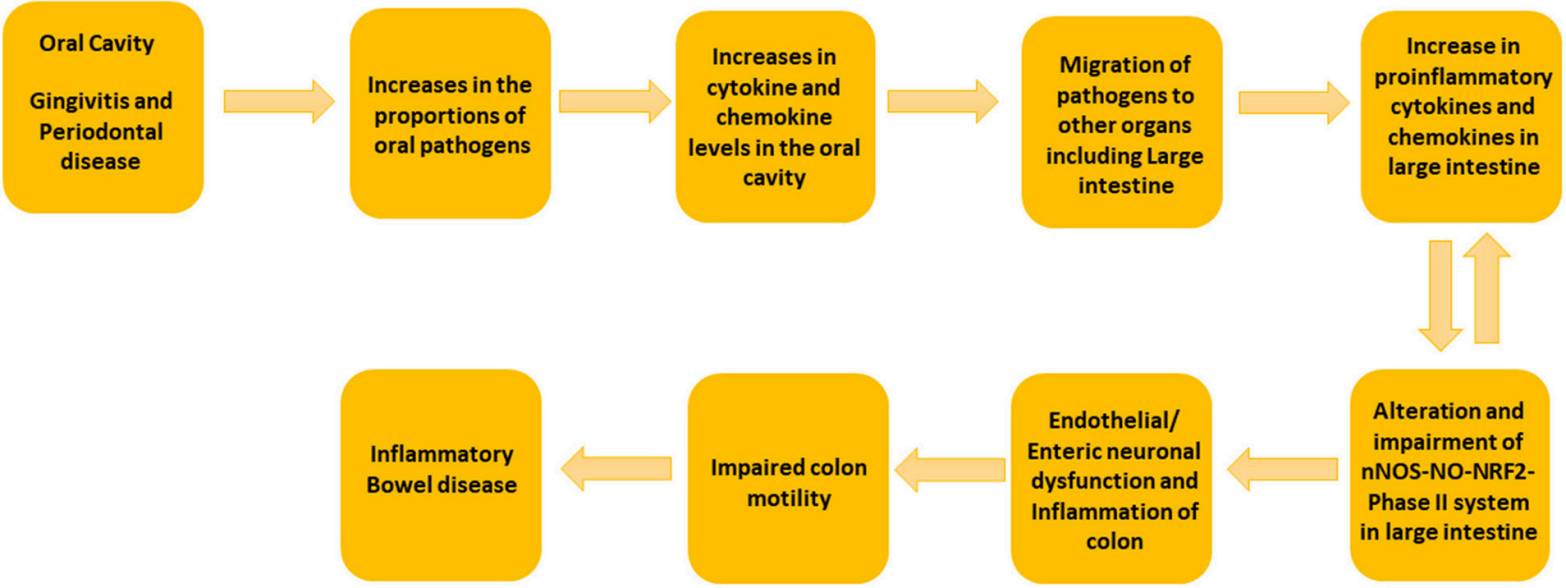
Schematic Representation of the suggested mechanism involved in the development of colitis by oral and gut microbiome. We propose that the increase in the concentrations of putative oral pathogens elevates the cytokine and chemokine levels in oral cavity. When putative oral pathogens travel to the gut, they can colonize locally and lead to the elevated levels of proinflammatory cytokines. This can effect on nNOS-NO-NRF2-Phase II system in the large intestine and could lead to colon dysmotility and colitis.

Oral bacterial species like *Porphyromonas, Peptostreptococcus, Eubacterium, Fusobacterium, Streptococcus salivarius, S. mitis, S. bovis, Veillonella* spp., *Staphylococcus* aureus, *S. epidermidis*, and *Campylobacter* spp. can convert nitrate to nitrite. A large amount of bioactive NO is found in the gastrointestinal tract, generated by dietary sources and by conversion of anaerobic bacteria in the oral cavity, or by anaerobic reaction with nitrate in the colon by *Escherichia coli* spp. The entero-salivary nitrate conversion pathway provides a rich source of bioactive NO and nitrate-reducing bacteria, such as *Veillonella*. In this pathway, nitrate is obtained by the salivary gland and is then concentrated in the saliva. Various facultative anaerobic bacteria on the top of the tongue effectively reduces nitrate to nitrite. The bacteria then use the nitrate and the nitrite as electron acceptors in their respiration process. This also helps the host in the first steps of converting nitrate to NO. The salivary nitrate then reaches the systemic circulation, various enzymatic reactions occur leading to reduction to NO, and other reactive nitrogen intermediates. The oral cavity plays an important role the production of nitric oxide, and specifically, employs the nitrate-nitrite-NO pathway in the oral cavity. It is well known that oral cavity bacteria can migrate to the colon. Taken together, our data suggest that the putative oral pathogens found in diseased colon specimens may survive by exploiting the nitrate-nitrite-NO pathway to modulate the proportion of non-detrimental gut bacteria, thus potentially worsening the condition of colon in colitis patients (Figure 4).

Previous studies suggest that enteric neurons and smooth muscle mediated gut motility is impaired in colitis patients (Snape et al., 1991; Vermillion et al., 1993). IBD associated gut inflammation affects the morphological and functional changes in the myenteric/enteric nervous system (ENS) and nitric oxide (NO) synthesis (Takahashi, 2003; Kono et al., 2004). Experimental studies have also shown that gut bacteria have a role in oxidative stress induced gut inflammation by controlling metabolic endotoxemia in obese mice (Cani et al., 2008). We have shown that polybacterial oral infection decrease the expression of nNOS and NRF2-phase II enzymes in the gut and this could lead to impaired colon motility (Gangula et al., 2015; Walker et al., 2018).

Some of the gut bacteria we have identified in the full thickness colon specimens in the present study, including *Bacteroides, Prevotella, Pseudomonas*, etc., have been identified in colon mucosal biopsies in earlier studies (Bibiloni et al., 2006). These bacteria evoke inflammatory responses affecting the innermost lining of colon. Many specific beneficial bacteria, including members of *Bacteroides* and *Prevotella* groups, *C. coccoides*, and Lactic acid bacteria were known to be decreased in colitis patients (Gibson et al., 1991). Specimens used in prior studies were colon mucosal biopsies or stool samples; but not full thickness colon specimens (Gibson et al., 1991; Bibiloni et al., 2006). Full-thickness colon consists of four layers of tissue including mucosa, submucosa, muscularis, and serosa.

Novel to this research design, full thickness colon specimens were obtained because colitis patients often experience colon motility abnormalities (Snape et al., 1991; Annese et al., 1997; Vrees et al., 2002). Several lines of evidence suggest that nitrergic neurons that releases NO via nNOS are known to play a pivotal role in colon motility (Kono et al., 2004; Winston et al., 2013). Previous studies have demonstrated that nitrergic neurons are degenerated in colitis (Onori et al., 2005; Sung et al., 2006). Recent studies from our laboratory indicate that nNOS, as well as antioxidants (NRF2 regulated-Phase II enzymes) protein expression are down-regulated in diseased colon specimens (Myers et al., 2014; Gangula et al., 2017). Furthermore, our previous studies demonstrated that polybacterial infection led to a decrease in nNOS, NRF2 and antioxidants protein expression in the colon tissues (Gangula et al., 2015). In addition, studies have shown that NO may play homeostatic role in gut inflammation (Kolios et al., 2004). Taken together, our data suggest that elevated levels of oral and gut pathogens in diseased colon full thickness specimens could contribute to impaired nNOS-NO-NRF2-Phase II system and colon motility abnormalities in IBD patients (Figure 4).

To our knowledge, our study is the first to report the presence of several microbiota of unknown function in IBD including *Micrococcus luteus*, Chloracidobacteria, *Arthrobacter, Propionicimonas, Paludibacter, Chryseobacterium, Calothrix*, and *Novosphingobium* (Table 5). These new microbiota members have not been identified in mucosal/fecal specimens in previous studies, suggesting that these bacteria are primarily colonized in the neuromuscular compartment. Additional studies are warranted to characterize the novel bacteria and investigate their specific role in colon motility and constipation in IBD patients.

**Table 5:** Proportions of bacterial species of unknown function colonized in full thickness colon of colitis patients.

**Sl. No**.	**Bacteria genus**	**Bacteria species**	**Proportion (%)**	**Bacteria phylum**	**Function in IBD**	**NCBI genome database link**
**ADJACENT HEALTHY COLON**						
1	Micrococcus	luteus	0.01	Actinobacteria	No role in IBD	X
2	Arthrobacter	s__	0.01	Actinobacteria	No role in IBD	BL
3	Propionicimonas	s__	0.01	Actinobacteria	No role in IBD	BN
4	Paludibacter	s__	0.01	Bacteroidetes	No role in IBD	BW
5	Chryseobacterium	s__	0.02	Bacteroidetes	No role in IBD	CF
6	Calothrix	s__	0.03	Cyanobacteria	No role in IBD	CK
7	Novosphingobium	s__	0.02	Proteobacteria	No role in IBD	FE
**DISEASED COLON (ULCERATIVE COLITIS)**						
1	Micrococcus	luteus	0.01	Actinobacteria	No role in IBD	X
2	Arthrobacter	s__	0.02	Actinobacteria	No role in IBD	BL
3	Propionicimonas	s__	0.1	Actinobacteria	No role in IBD	BN
4	Paludibacter	s__	0.03	Bacteroidetes	No role in IBD	BW
5	Chryseobacterium	s__	0.1	Bacteroidetes	No role in IBD	CF
6	Calothrix	s__	0.1	Cyanobacteria	No role in IBD	CK
7	Novosphingobium	s__	0.04	Proteobacteria	No role in IBD	FE
**DISEASED COLON (CROHN'S COLITIS)**						
1	Micrococcus	luteus	0.02	Actinobacteria	No role in IBD	X
2	Arthrobacter	s__	0.2	Actinobacteria	No role in IBD	BL
3	Propionicimonas	s__	0.02	Actinobacteria	No role in IBD	BN
4	Paludibacter	s__	0.02	Bacteroidetes	No role in IBD	BW
5	Chryseobacterium	s__	0.3	Bacteroidetes	No role in IBD	CF
6	Calothrix	s__	0.1	Cyanobacteria	No role in IBD	CK
7	Novosphingobium	s__	0.01	Proteobacteria	No role in IBD	FE

*Specific Information of functions was adapted from NCBI Genome Database (https://www.ncbi.nlm.nih.gov/genome/)*.

*The bacterial species that could not be identified at the genus level are mentioned as g___ and the bacterial species that could not be identified at the species level are mentioned as s___*.

In summary, this study have identified specific bacterial pathogens potentially associated with colon motility in IBD patients. The observations showed that some putative oral pathogens belonging to the Phyla Firmicutes (Streptococcus, Staphylococcus, Peptostreptococcus), and Fusobacteria (Fusobacterium) dominated in the microbiomes of CC and UC diseased specimens and might involve the modulation of colon motility in IBD.

## Study Limitations

The limitations of the study include the smaller sample size across disease and race groups making this as a preliminary study. In spite of the limitations in sample size and the fact that some of the identified bacteria were not significantly altered in colitis specimens, we were still able to observe differences in the microbiome between CA and AA colitis patients. This could be due to amplicon sequencing of a shorter conserved region of 16S rRNA gene instead of in depth shotgun sequencing. Moreover, we did not profile the oral microbiome from oral specimens (dental plaque, etc.) in the same IBD patients from whom full thickness colon specimens were collected. Finally, host-microbiome interaction studies are needed to better discern specific roles of the oral and gut bacteria in the development of colitis. Future studies are aimed to collect oral and fecal specimens therefore a comparative experiments in regards to changes in microbiome, along with specific key proteins will be conducted from the same patient.

## Author Contributions

VD, SM, KS, SP, SS, PG, and MT have contributed both for data analysis and manuscript preparation. DS, CF-D, LK, SA, and JS have contributed in manuscript preparation.

### Conflict of Interest Statement

The authors declare that the research was conducted in the absence of any commercial or financial relationships that could be construed as a potential conflict of interest.
